# Predictive Processing Over the Course of Aging: Multiple Timescales of Effective Connectivity

**DOI:** 10.1111/ejn.70387

**Published:** 2026-01-07

**Authors:** Martin Tom Banaschewski, Christoph Mathys, István Winkler, Juanita Todd, Ryszard Auksztulewicz

**Affiliations:** ^1^ Department of Education and Psychology Freie Universität Berlin Berlin Germany; ^2^ Interacting Minds Centre Aarhus University Denmark; ^3^ Institute of Psychology and Cognitive Neuroscience HUN‐REN Research Centre for Natural Sciences Budapest Hungary; ^4^ School of Psychological Science University of Newcastle Newcastle Australia; ^5^ Department of Neuropsychology and Psychopharmacology Maastricht University Maastricht the Netherlands

**Keywords:** aging, connectivity, EEG, learning, MMN

## Abstract

Predictive processing theories describe perception as a dynamic interplay between top‐down predictions and bottom‐up prediction errors across hierarchical stages of sensory processing. However, it remains unclear how neural connectivity flexibly adapts to changing sensory environments over time, and how these dynamics are influenced by aging. This study investigated how temporal factors on three distinct timescales, as well as age, shape neural responses and connectivity to dynamically changing auditory stimuli. Electroencephalography (EEG) data were recorded from 63 participants aged 18–75 as they listened to sequences of tones, where rare and unexpected “original deviants” became standards over time, and previously standard tones became “reverse deviants.” Event‐related potentials (ERPs) were more pronounced for original deviants than reverse deviants. Amplitudes increased on short timescales (seconds) but declined over longer timescales (minutes) and with advancing age. To infer the neural mechanisms underlying these effects, dynamic causal modelling (DCM) was used to analyze effective connectivity. DCM revealed increased descending (top‐down) connectivity for original deviants, consistent with a stronger reliance on predictions. Additionally, intrinsic (within‐region) connectivity increased over seconds but decreased over minutes, reflecting timescale‐dependent neural adaptation. Aging was associated with stronger modulation of descending connectivity by deviant type but weaker modulation by slow dynamics. These results underscore the brain's ability to dynamically adapt to changing sensory environments at multiple timescales and for the first time reveal age‐related changes in the dynamics of this adaptation.

## Graphical abstract

This study used electroencephalography (EEG) and dynamic causal modelling to examine how predictive auditory processing and neural connectivity adapt over time and with age. Sixty‐three participants (ages 18–75) listened to tone sequences where deviant and standard roles reversed between contexts, probing prediction at multiple timescales (seconds to minutes). The design allowed analysis of ERPs and connectivity changes linked to context effects, multi‐timescale dynamics, and aging.

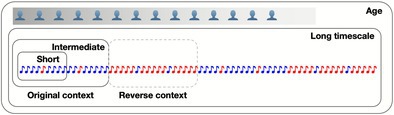

**Abbreviations**
A1: primary auditory cortex
BMA: Bayesian model averaging
BMR: Bayesian model reduction
DCM: dynamic causal modelling
EEG: electroencephalography
ERP: event‐related potential
FWE: family‐wise error
IFG: inferior frontal gyrus
MMN: mismatch negativity
MoCA: Montreal Cognitive Assessment
PEB: parametric empirical Bayes
PP: predictive processing
STG: superior temporal gyrus

## 1 Introduction

Perceiving a dynamic and complex sensory environment is challenged by contextual uncertainty (A. Clark 2013; Hohwy 2020). Sensory inputs that appear familiar and predictable in one context may seem rare or unexpected in another. This challenge lies at the heart of the inference problem: accurately identifying the environmental causes of sensory inputs (K. Friston et al. 2015). Predictive processing (PP) models propose that perception emerges from hierarchical interactions between ascending and descending neural signals across different brain regions. In this framework, descending signals encode prior beliefs or predictions—internal models shaped by prior experience—while ascending signals carry prediction errors that prompt updates to these models (A. Clark 2013; Hohwy 2020). Both priors and prediction errors are represented as probability densities, with the width of each density reflecting the degree of uncertainty, or inverse precision.

Perceptual learning is often studied using the oddball paradigm, in which frequently occurring tones establish regularities and expectations, while infrequent deviant tones violate these expectations and elicit a mismatch negativity (MMN) response in electroencephalography (EEG) recordings (May 2021; Näätänen et al. 2011). The classical oddball paradigm has undergone several adaptations (Bendixen et al. 2012; Näätänen et al. 2019), including the multi‐timescale oddball paradigm (Todd et al. 2011), which enables a more nuanced investigation of the temporal dynamics underlying perceptual learning. In this paradigm, two tones alternate between standard (frequent) and deviant (rare) roles across blocks, allowing researchers to examine how initial role assignment continues to influence neural responses despite probability reversals (Costa‐Faidella et al. 2011). Event‐related potential (ERP) studies have demonstrated that these initial assignments shape subsequent MMN responses at the sensor level—a phenomenon known as the *primacy effect* (Frost et al. 2016; Todd et al. 2021). This effect has been robustly replicated across various participant samples and sequence types (Fitzgerald et al. 2018; Frost et al. 2016; Todd et al. 2014, 2021). Beyond block‐level reversals, perceptual learning unfolds across multiple timescales. Short‐term adaptation can occur over seconds (Winkler, Karmos, and Näätänen 1996; Garrido, Kilner, Kiebel, Stephan, et al. 2009; Lieder et al. 2013), while longer‐term learning effects—such as persistent biases in MMN responses—have been observed throughout experimental sessions over several minutes (Fitzgerald and Todd 2018). These findings suggest that the auditory system integrates contextual information over both transient and sustained durations (Fitzgerald and Todd 2018; Kiebel et al. 2008), likely reflecting distinct neural mechanisms operating at different levels of the perceptual hierarchy.

Beyond contextual factors, aging has a significant impact on perceptual learning and auditory change detection, as evidenced by changes in MMN. Numerous studies have demonstrated that the amplitude of MMN is reduced in older adults, suggesting diminished sensitivity to auditory deviance (Näätänen et al. 2012; Cheng et al. 2013) independent of age‐related hearing loss (Brückmann et al. 2021; Boncz et al. 2024). Recent work shows that this decline may reflect age‐related reduction in the automatic encoding and updating of auditory regularities—especially at longer timescales—rather than general memory deficits (Todd et al. 2022). However, age‐related reductions in MMN amplitude have been shown to predict more specific mnemonic deficits related to memory precision, particularly in the ability to discriminate similar past stimuli (Chow et al. 2025)

Neural mechanisms underlying ERP differences (such as MMN) can be inferred using computational methods such as effective connectivity analyses (Gütlin et al. 2025). Effective connectivity offers insights into the roles of ascending (bottom‐up), descending (top‐down), and intrinsic (gain‐modulatory) pathways in the processing of deviant auditory stimuli, as well as how these pathways are affected by contextual factors (e.g., the primacy effect) or inter‐individual differences (e.g., age). Dynamic causal modelling (DCM) is a prominent technique for inferring effective connectivity from noninvasive neuroimaging data such as EEG time‐series, within a principled, model‐based framework (K. J. Friston et al. 2003; Kiebel et al. 2006; Moran, Pinotsis, and Friston 2013). By integrating Bayesian inference with biologically plausible models of neural dynamics, DCM enables the testing of mechanistic hypotheses about how predictive coding processes—specifically, predictions, prediction errors, and their precision—influence connectivity patterns across time. This makes DCM particularly well‐suited for studying the temporal and hierarchical dynamics of PP.

Previous DCM studies have associated MMN generation with changes in effective connectivity between auditory and frontal cortical regions (Garrido et al. 2008; Garrido, Kilner, Kiebel, and Friston 2009), reflected in ascending connections (thought to mediate prediction errors) as well as in descending connections (interpreted as putative prediction updates due to prediction error signals). A more recent MMN study directly focusing on effective connectivity modulations across contexts showed that tones initially presented as deviants, compared to tones which become deviants later in the sequence, show greater modulation of descending connections from higher to lower auditory regions, as well as higher precision of intrinsic connections (Fitzgerald et al. 2021). This finding suggested that the primary effect may be mediated by stronger neural activity involved in prediction updates. However, studies investigating the effect of age on effective connectivity underlying MMN yielded heterogeneous results: while one study (Moran et al. 2014) has reported an age‐related decrease of the ascending connection from lower to higher auditory region, another study has found age‐related reductions of intrinsic connectivity in the inferior frontal gyrus (Cooray et al. 2014). Both these studies were based on a classical oddball paradigm, where tone assignment to deviant versus standard is fixed for the entire recording session.

The current study expands on previous research by analyzing and modelling EEG data recorded in a multi‐timescale deviant sequence presented to participants across a wide age range. Specifically, we examine how neural connectivity adapts to contextual effects (primacy), reflects dynamics over three different timescales, and is modulated by aging.

## 2 Materials and Methods

### 2.1 Participants

A total of 63 participants (46 female; aged 18–75 years, mean = 47.7, SD = 20.6) were recruited from the student bodies of the University of Newcastle and a community group known as the University of the Third Age, both in Newcastle (Australia). Exclusion criteria included a history of neurological or psychiatric disorders, cognitive impairment, prior head injuries, brain surgeries, and a diagnosis of schizophrenia or first‐degree relatives with schizophrenia. Hearing was assessed using an audiometer as a screening tool. All participants except five had normal hearing. Among the five remaining participants, three had hearing thresholds within the “slight” hearing loss range (25 dB) and two within the “mild” range (35 and 40 dB) based upon previously established criteria (J. G. Clark 1981). In exchange for their participation, subjects either received course credit or monetary compensation. The study was approved by the Human Research Ethics Committee of the University of Newcastle (H‐2012‐0270).

### 2.2 Procedure

Before the experiment, participants underwent a hearing threshold assessment using a pure tone audiometer (Earscan ES3S) and cognitive screening via the Montreal Cognitive Assessment (MoCA) (Nasreddine et al. 2005) for participants aged 40 and older to ensure eligibility. During the experiment, participants' EEG activity was recorded while they passively attended auditory stimuli. They wore electrode caps and Sennheiser HD280 Pro stereo headphones. A silent subtitled DVD was presented to maintain engagement throughout the experiment. Participants were instructed to minimize movement and ignore the auditory stimuli.

### 2.3 Experimental Stimuli

**FIGURE 1:**
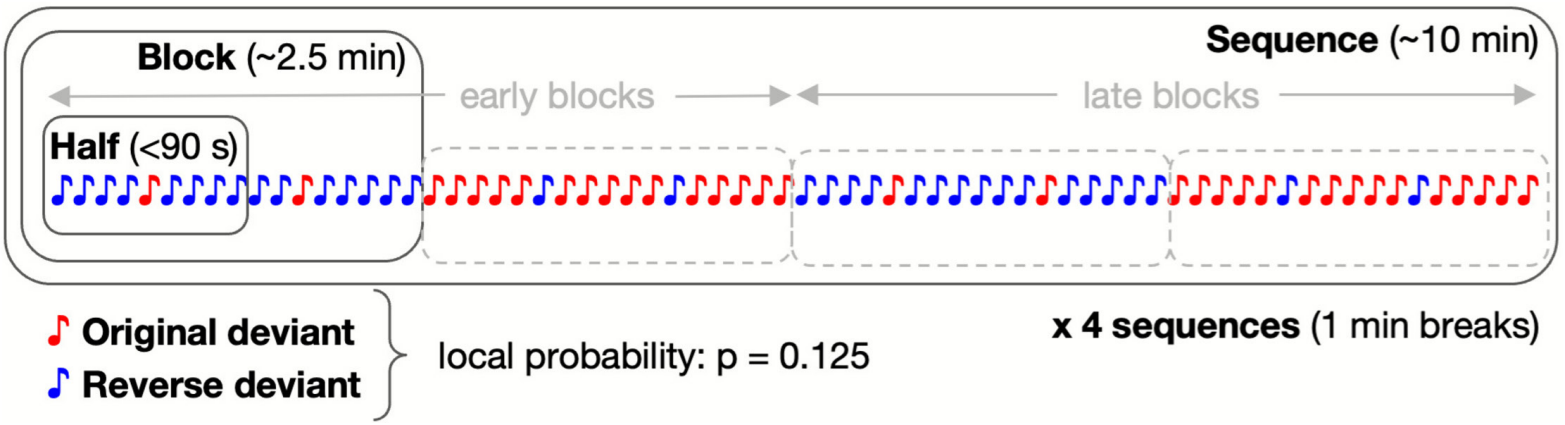
Multi‐timescale paradigm. The whole experiment lasted for about 42 min and contained four identical sequences (only one is displayed) with in‐between breaks of 1 min. Each sequence consisted of four blocks in which pure tones, with durations of 30 ms or 60 ms, were presented at imbalance rates of occurrence, with a stimulus onset asynchrony of 300 ms. Within the first and third block of each sequence, the 30 ms tone served as a local deviant (p = 0.125), while the 60‐ms tone was the local standard (p = 0.875). For the second and fourth block of each sequence, local tone probabilities were inverted, such that the 60‐ms tone served as the deviant stimulus and the 30‐ms tone as standard. For analysis of effects at the short time scale, blocks were further subdivided into halves.

A multi‐timescale auditory oddball paradigm was used, featuring two pure tones (1000 Hz, 75 dB, rise and fall times each 5 ms) of different durations (30 ms, 60 ms) that alternated as frequent standards (*p* = 0.875) and rare deviants (*p* = 0.125) across 16 blocks, arranged in four identical sequences. Tone roles were reversed after each block through inversion of their probabilities. Each block contained 420 standards and 60 deviants. The experiment lasted approximately 42 min, with 1‐min breaks between sequences. Figure 1 visualizes one of four identical sequences from the experiment's multi‐timescale structure. The paradigm allowed examination at three different timescales. At the shortest timescale, differences between block halves were compared. The intermediate timescale contrasted instances of homologous blocks. At the longest timescale, different sequences were contrasted. Stimuli were presented binaurally with a 300‐ms onset asynchrony, and at least three standards always preceded each deviant.

### 2.4 Recording and Preprocessing of EEG Data

EEG was recorded using a Neuroscan Quikcap with 64 Ag/AgCl scalp electrodes and a Synamps 2 system (1000‐Hz sampling rate, referenced to the nose). Preprocessing included downsampling (300 Hz), high‐pass filtering (0.01 Hz), low‐pass filtering (90 Hz), notch filtering (48–52 Hz), eyeblink correction based on removing the top two principal components related to the average eyeblink (Ille et al. 2002), re‐referencing to the common average, epoching (−50 to 250 ms), baseline correction, robust averaging, and final low‐pass filtering (40 Hz) applied to remove high‐frequency artefacts which may have possibly been introduced during robust averaging (Litvak et al. 2011). The specific step of re‐referencing to the common average is a prerequisite for subsequent DCM analysis (Litvak et al. 2011) and differs from non‐DCM studies using the same types of auditory sequences (Fitzgerald et al. 2018; Todd et al. 2021, 2022).

### 2.5 Sensor Space Analysis

ERPs were analyzed using multiple regression to confirm significant effects at the sensor level before investigation with DCM. Four within‐subject factors were included in the analysis. Deviant type was bifactorial (factor levels: 1. original, 2. reversed) and represented whether a tone was a deviant at first encounter or became one after reversion. Block half was also bifactorial (1. first, 2. second) and represented the shortest timescale. The intermediate timescale was represented by block number (bifactorial: 1. early, 2. late). The longest timescale was represented by sequence number which had one factor level for each sequence (1st, 2nd, 3rd, 4th). At the between‐subjects level, the (linear) age factor was z‐scored and included in the general linear model. Significant main effects and interactions were tested using family‐wise error (FWE) corrected cluster‐based analysis. Statistical parametric maps were thresholded at *p* < 0.005 and corrected at the cluster‐level *p*_FWE_ < 0.05. Significant effects from the sensor space analysis were passed on to DCM in order to shed light on the underlying changes in effective connectivity of the brain.

In an additional analysis, to model a potential nonlinear relationship between age and the outcome, we included both a linear term (z‐scored age) and a nonlinear term derived by taking the absolute value of the z‐scored age and then z‐scoring the result. This transformation captures the magnitude of deviation from the mean age, regardless of direction, and allows for the modelling of symmetric, U‐shaped effects relative to mean age. The remaining analysis steps and statistical threshold were kept identical to the main analysis.

### 2.6 Dynamic Causal Modelling

DCM is a computational approach used to infer effective connectivity between brain regions and to determine how experimental conditions influence these connections (Kiebel et al. 2006). Unlike conventional EEG analyses, which describe observed neural activity, DCM models the causal relationships between brain regions by estimating the underlying neural dynamics. By integrating Bayesian inference with biologically plausible neural models, DCM allows testing hypotheses about the mechanisms that drive observed responses.

A dynamic causal model is both biophysical and generative. As a biophysical model, DCM incorporates realistic assumptions about neural connectivity, treating the brain as a network in which activity in one region influences activity in another. Each modelled brain region is further divided into cortical layers that contain pyramidal cells, inhibitory interneurons, and spiny‐stellate cells. These neural populations interact in accordance with well‐established neurophysiological principles (Felleman and Van Essen 1991; Jansen and Rit 1995).

As a generative model, DCM generates synthetic neural data by estimating hidden parameters that cannot be directly observed. These parameters represent connectivity strengths and their condition‐dependent modulation. Through an iterative process, DCM refines these parameters to maximize the likelihood that the synthetic data match the empirically recorded EEG responses (Zeidman, Jafarian, Corbin, et al. 2019).

The process of DCM consists of several key steps that allow for the estimation of neural interactions. First, experimental stimuli, such as auditory deviants, are specified as driving inputs, which evoke neural activity in primary sensory areas. In addition to these driving inputs, DCM also accounts for modulatory inputs, which describe condition‐specific influences on connectivity. Following input specification, the next step involves selecting the brain regions to be included in the model. These regions can be chosen based on prior literature or identified statistically through EEG source reconstruction. The selected regions are then incorporated into a system of differential equations that define their interactions.

To estimate effective connectivity, DCM partitions neural dynamics into three key matrices, each representing a different aspect of connectivity. The A matrix describes the baseline effective connectivity between and within brain regions, which remains constant regardless of experimental conditions. The B matrix specifies condition‐dependent changes in connectivity, allowing the model to quantify how experimental factors influence neural interactions. The C matrix represents the influence of external stimuli, such as auditory deviants, on connection strengths (Zeidman, Jafarian, Corbin, et al. 2019).

Once these matrices are defined, model inversion is performed using Bayesian inference. This process involves combining prior parameter distributions with empirical EEG data to estimate posterior distributions that best explain the observed neural responses. Prior to model inversion, sensor‐level data were projected onto the top eight principal eigenvectors, reducing dimensionality while retaining the components that capture the most variance in the signal. To balance model complexity and accuracy, DCM uses negative variational free energy as an approximation of model evidence (K. Friston et al. 2006). This ensures that the selected model provides the most parsimonious explanation of the data.

To generalize DCM findings across participants, parametric empirical Bayes (PEB) is applied at the group level (Zeidman, Jafarian, Seghier, et al. 2019). PEB models the variability in individual DCMs by decomposing connectivity parameters into fixed effects, which reflect commonalities across participants, and random effects, which capture individual differences. By incorporating Bayesian model reduction (BMR), PEB efficiently evaluates reduced models and identifies the best‐fitting explanation for group‐level connectivity changes. The final group‐level estimates are obtained using Bayesian model averaging (BMA), which optimally weighs individual models based on their explanatory power.

In the present study, DCM was used to investigate how effective connectivity adapts to auditory deviants across multiple timescales. Experimental EEG data was analyzed to model neural interactions between the primary auditory cortex (A1), superior temporal gyrus (STG), and inferior frontal gyrus (IFG) in both hemispheres. Cortical localization was based on the coordinates provided by the Montreal Neurological Institute (Garrido, Kilner, Kiebel, and Friston 2009): left A1 (−42, −22, 7), right A1 (46, −14, 8), left STG (−61, −32, 8), right STG (59, −25, 8), left IFG (−46, 20, 8), right IFG (46, 20, 8). These cortical sources, as well as the DCM of the auditory deviant response, have been adopted in several previous studies (Fitzgerald et al. 2021; Phillips et al. 2016; Rosch et al. 2019; López‐Caballero et al. 2025).

The model space included 4096 different DCMs per participant, allowing for the examination of four main effects: deviant type (original vs. reversed), block half (first vs. second), block number (early vs. late), and sequence number (1st, 2nd, 3rd, and 4th sequence). Connectivity was analyzed within three distinct connectivity groups: bottom‐up connections (e.g., A1 to STG, STG to IFG), which reflect prediction‐error signaling; top‐down connections (e.g., IFG to STG, STG to A1), which indicate the influence of priors; and intrinsic connectivity (self‐connections within A1, STG, and IFG), which regulate precision weighting. Each of the three connectivity groups could be switched “on” (i.e., subject to modulation by a given effect) or “off” (i.e., independent of the effect) for each of the four effects, amounting to 2^3*4^ = 4096 models.

For each participant, a full model was initially inverted, allowing all connections to be modulated by all experimental factors. BMR was then applied to derive the most parsimonious model, which was subsequently analyzed using PEB at the group level. The final model selection was based on negative variational free energy, ensuring an optimal trade‐off between model complexity and accuracy.

By linking observed EEG responses to underlying connectivity dynamics, this study aims to clarify how the brain updates internal models in response to changing auditory environments. The results provide insight into PP mechanisms, particularly how neural connectivity evolves over time in response to stimulus exposure.

## 3 Results

### 3.1 Sensor Space Analysis

ERPs in response to deviant tones followed the characteristic pattern, with early electrical negativity in posterior regions (peak 60 ms after stimulus onset over channel P7, *T*_62_ = 12.61, *p*_FWE_ < 0.001) and positivity in frontal regions (peak 57 ms after stimulus onset over channels FC1 and FC2; *T*_62_ = 13.07, *p*_FWE_ < 0.001), followed by an inverse pattern at late latencies (posterior positivity: peak 180 ms after stimulus onset over channel TP7, *T*_62_ = 4.11, *p*_FWE_ < 0.001; frontal negativity: peak 180 ms after stimulus onset over channel FC2, *T*_62_ = 8.20, *p*_FWE_ < 0.001). Throughout the next sections, we refer to any response latencies shorter or equal to 100 ms as “early” and to longer latencies as “late.”

#### 3.1.1 Effects of Deviant Type

**FIGURE 2:**
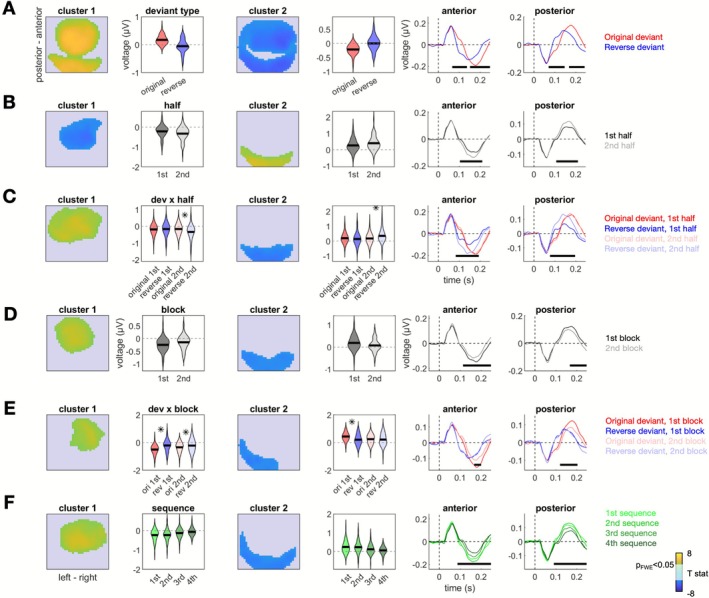
ERP results (independent of age). (A) Main effects of deviant type (original vs. reversed). Topographic maps show significant clusters, averaged over time. Cluster 1 shows more positive (peak 100 ms poststimulus over anterior channels) or less negative (peak 200 ms over posterior channels) ERP amplitudes, while cluster 2 shows more negative (peak 97 ms over posterior channels) or less positive (peak 203 ms over anterior channels) ERP amplitudes, for original versus reverse deviants. Violin plots show the group distribution of ERP voltage values, averaged per participant and condition over each significant cluster. ERP plots show average deviant‐evoked waveforms sorted by condition, separately for each significant cluster. Bold horizontal lines denote significant differences between conditions (*p*_FWE_ < 0.05). (B) Main effects of the short timescale (1st vs. 2nd half). Legend as above. Cluster 1 shows more negative (peak 120 ms over anterior channels) ERP amplitudes, while cluster 2 shows more positive (peak 123 ms over posterior channels) ERP amplitudes, for reverse vs. original deviants in the 2nd but not in the 1st half. (C) Interaction between deviant type and the short timescale. Cluster 1 shows less negative (peak 167 ms over anterior channels) ERP amplitudes, while cluster 2 shows less positive (peak 167 ms over posterior channels) ERP amplitudes, for 1st versus 2nd half. Legend as above. Asterisks denote post hoc *p* < 0.05. (D) Main effects of the intermediate timescale (1st vs. 2nd block). Cluster 1 shows more negative (peak 220 ms over anterior channels) ERP amplitudes, while cluster 2 shows more positive (peak 230 ms over posterior channels) ERP amplitudes, for 1st versus 2nd block. Legend as above. (E) Interaction between deviant type and the intermediate timescale. Cluster 1 shows more positive (peak 190 ms over anterior channels) ERP amplitudes, while cluster 2 shows more negative (peak 187 ms over posterior channels) ERP amplitudes, for original vs. reverse deviants in the 1st block, and less pronounced differences in the 2nd block. Legend as above. (F) Main effects of the long timescale (1st vs. 2nd vs. 3rd vs. 4th sequence). Cluster 1 shows less negative (peak 117 ms over anterior channels) ERP amplitudes, while cluster 2 shows less positive (peak 233 ms over posterior channels) ERP amplitudes, for later versus earlier sequences. Legend as above.

Comparing deviant types, original deviants evoked stronger and more temporally extended ERPs than reverse deviants (early frontal: peak 100 ms after stimulus onset over channel FC1, *T*_62_ = 7.27, *p*_FWE_ < 0.001; early posterior: peak 97 ms after stimulus onset over channel P8, *T*_62_ = 7.03, *p*_FWE_ < 0.001; late frontal: peak 203 ms after stimulus onset over channel FC4, *T*_62_ = 7.40, *p*_FWE_ < 0.001; late posterior: peak 200 ms after stimulus onset over channel PO5, *T*_62_ = 6.70, *p*_FWE_ < 0.001). This effect is visualized in Figure 2A, showing the relative ERP response to the original compared to the reversed deviant.

#### 3.1.2 Effects of Timescales

At the shortest timescale, ERP amplitudes increased in the second half of blocks compared to the first half, but only at late latencies (late frontal: peak 167 ms after stimulus onset over channel FC1, *T*_62_ = 6.01, *p*_FWE_ < 0.001; late posterior: peak 167 ms after stimulus onset over channel PO6, *T*_62_ = 5.47, *p*_FWE_ = 0.002). This main effect of block half is depicted in Figure 2B. Furthermore, this effect interacted with deviant type at late latencies in frontal (peak 120 ms after stimulus onset over channel F1, *T*_62_ = 5.82, *p*_FWE_ < 0.001) and posterior (peak 123 ms after stimulus onset over channel O1, *T*_62_ = 6.01, *p*_FWE_ < 0.001) clusters (Figure 2C). A visual inspection of the interaction effect revealed that original deviants evoked stronger ERPs than reverse deviants already in the first half of each block, but this difference gradually diminished in the second half of each block, whereby the reverse deviant‐evoked ERP amplitudes approached those of the original deviants. Post hoc tests revealed that a significant difference between deviant types was found in the second half of each block (frontal: *T*_62_ = 4.26, *p* < 0.001; posterior: *T*_62_ = 3.96, *p* < 0.001), while no difference between reverse versus original deviant was found in the first half (frontal: *T*_62_ = −0.69, *p* = 0.48; posterior: *T*_62_ = −1.58, *p* = 0.13).

At the intermediate timescale, ERP amplitudes decreased across blocks. Specifically, there was a main effect of block number at late latencies (late frontal: peak 220 ms after stimulus onset over channel FC1, *T*_62_ = 5.46, *p*_FWE_ < 0.001; late posterior: peak 230 ms after stimulus onset over channel CP5, *T*_62_ = 4.64, *p*_FWE_ < 0.001) that indicated a reduced ERP amplitude to deviants in the late block compared to deviants in the early block of a sequence. Positive and negative main effects of block number on ERP response are depicted in Figure 2D. Again, this was accompanied by an interaction between the effect of block number and deviant type (late frontal: peak 190 ms after stimulus onset over channel FC4, *T*_62_ = 5.04, *p*_FWE_ = 0.006; late posterior: peak 187 ms after stimulus onset over channel P5, *T*_62_ = 5.00, *p*_FWE_ = 0.004). The interaction effect is visualized in Figure 2E and shows that while ERPs evoked by original deviants decrease over blocks, ERPs evoked by reverse deviants are stable. Post hoc tests of the interaction effect revealed that the primacy effect (i.e., stronger amplitudes for original vs. reverse deviants) was more pronounced in early blocks (frontal cluster: *T*_62_ = 7.05, *p* < 0.001; posterior cluster: *T*_62_ = 5.34, *p* < 0.001) than in late blocks, where it was only significant in the frontal cluster (frontal cluster: *T*_62_ = 2.34, *p* = 0.023; posterior cluster: *T*_62_ = 0.55, *p* = 0.58). This suggests a partial adaptation of the primacy effect at the intermediate timescale.

At the longest timescale, ERP responses to deviants from later sequences (separated by short breaks) were significantly weaker than those from earlier sequences. The difference was evident in frontal/central (peak 117 ms after stimulus onset over channel C1, *T*_62_ = 5.62, *p*_FWE_ < 0.001) and posterior (peak 233 ms after stimulus onset over channel O2, *T*_62_ = 5.38, *p*_FWE_ < 0.001) clusters at late latencies. Positive and negative main effects of sequence number on ERP response are depicted in Figure 2F. There was no evidence of an interaction between sequence number and deviant type, suggesting that the primacy effect was stable at the longest timescale (i.e., over the 4 sequences separated by short breaks).

#### 3.1.3 Linear Effects of Age

**FIGURE 3:**
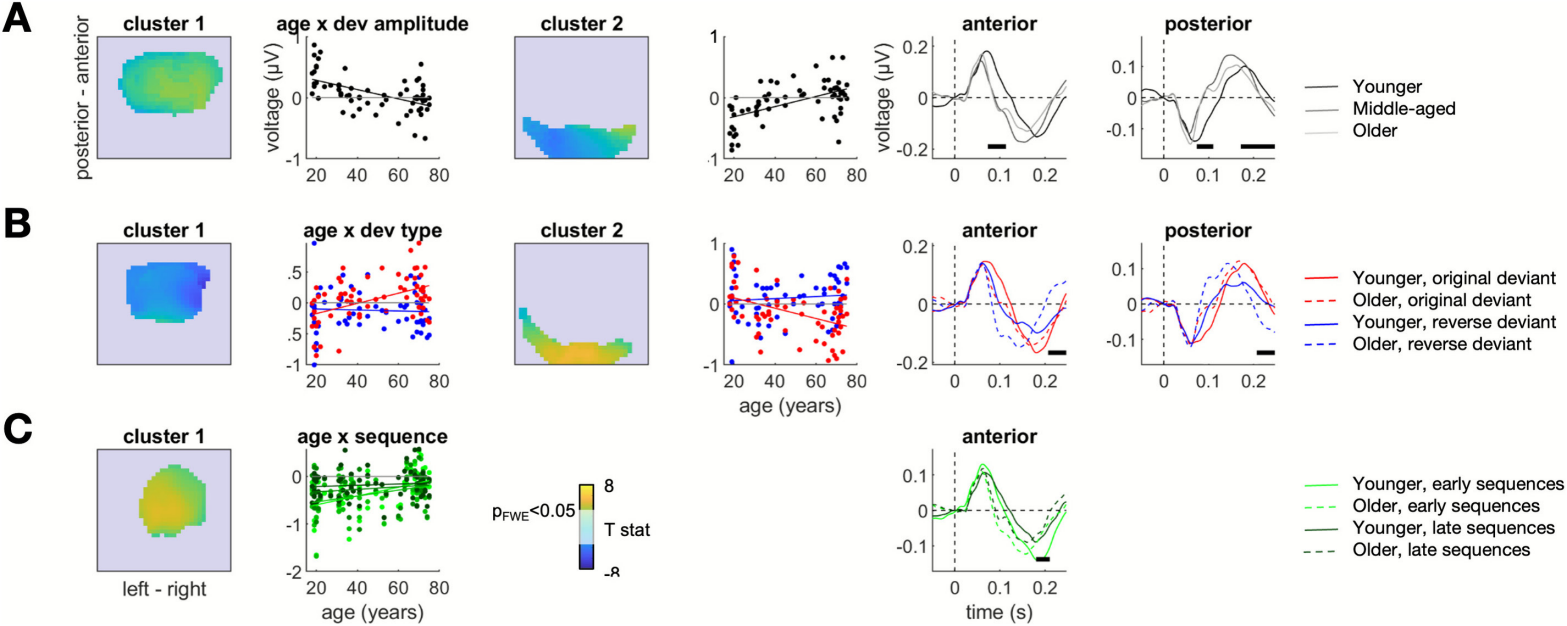
ERP results (effects of age). (A) Age effects on deviant‐evoked amplitude (independent of deviant type or timescale). Topographic maps show two significant clusters, averaged over time. Scatterplots with regression lines show the age effect on ERP voltage values, averaged per participant and condition over each significant cluster. Asterisks denote *p*_FWE_ < 0.05. ERP plots show average deviant‐evoked waveforms sorted by condition, separately for each significant cluster (anterior vs. posterior electrodes). (B) Interaction between age and deviant type (original vs. reverse). Legend as above. (C) Interaction between age and the long timescale (1st vs. 2nd vs. 3rd vs. 4th sequence). Legend as above. Only one significant cluster was identified.

At the between‐subjects level, assuming a linear effect of age, older participants showed a decreased amplitude of ERP response at early latencies (linear regression with age as a continuous regressor; anterior: peak 90 ms after stimulus onset over channel F3, *T*_62_ = 5.59, *p*_FWE_ = 0.003; posterior: peak 87 ms after stimulus onset over channel Oz, *T*_62_ = 5.54, *p*_FWE_ = 0.034). A visual inspection of this result (Figure 3A) indicated that age‐related ERP amplitude reduction corresponds to a faster return of the ERP amplitude to the baseline following an initial peak.

Furthermore, age interacted with the effect of deviant type (Figure 3B). Specifically, a significant interaction between deviant type and participant age could be found at late latencies (anterior: peak 240 ms after stimulus onset over channel FC4, *T*_62_ = 4.35, *p*_FWE_ = 0.004; posterior: peak 230 ms after stimulus onset over channel O1, *T*_62_ = 4.40, *p*_FWE_ = 0.023). While the regression slopes indicate more pronounced reverse deviant‐evoked ERP amplitudes for older participants, a visual inspection of the ERPs indicates that this is largely due to the reverse deviant‐evoked ERP having an earlier peak followed by an additional rebound, as compared to the original deviant‐evoked ERP in older participants as well as both ERPs in younger participants.

Finally, we found a significant interaction between the longest time scale (sequence number) and age (Figure 3C), expressed over anterior/central channels at late latencies (peak 193 ms after stimulus onset over channel C1, *T*_62_ = 4.13, *p*_FWE_ = 0.013). This effect revealed that the reduction of deviant‐evoked ERP amplitudes over the longest timescale was more pronounced in younger than in older participants.

The remaining main effects (short and intermediate time scales) and the interactions of time scales with deviant type were not further modulated by age (all *p*_FWE_ > 0.05).

#### 3.1.4 Nonlinear Effects of Age

The results of an additional analysis, in which a nonlinear (U‐shaped) age factor was included next to the linear effect reported above, revealed that age exerted a nonlinear effect on the overall ERP response amplitude (anterior: peak 133 ms after stimulus onset over channel C2, *T*_62_ = 4.03, *p*_FWE_ = 0.004; posterior: peak 127 ms after stimulus onset over channel P2, *T*_62_ = 4.19, *p*_FWE_ = 0.011). However, the effects of deviant type and the three time scales, as well as interactions between them, were not modulated by age in a nonlinear manner (all *p*_FWE_ > 0.05). As a result, only the linear effects of age were included in subsequent DCM analysis.

### 3.2 Dynamic Causal Modelling

The DCM analysis was split into two parts. First, we analyzed the effects of deviant type and the three distinct time scales independent of age. In this case, the full model space consisted of 4096 models per participant, which were analyzed using BMR and parametric empirical Bayes (PEB) to identify the best explanation for the observed ERP differences. The winning model (model probability *p* = 0.98 out of all 4096 models) allowed for a subset of connections to be modulated by short and intermediate time scales, and all connections to be modulated by deviant type and the long time scale. Table 1 provides a full summary of all parameters at the group level. Modulation values under an experimental condition can be interpreted as connection strength in percent relative to its value under the opposed experimental condition.

**TABLE 1:** Effects of deviant type and multiple time scales on DCM connectivity estimates.

Effect	Connection type	From	To	Posterior mean	Variance (×10^−3^)
Deviant type (original vs. reverse)	Intrinsic	Left A1	Left A1	−0.0689	0.131
		Right A1	Right A1	−0.1442	0.1403
		Left STG	Left STG	0.1056	0.1486
		Right STG	Right STG	0.1077	0.1393
		Left IFG	Left IFG	0.0151	0.1574
		Right IFG	Right IFG	0.0678	0.1414
	Ascending	Left A1	Left STG	−0.2112	0.2871
		Right A1	Right STG	−0.0797	0.2853
		Left STG	Left IFG	−0.1257	0.3945
		Right STG	Right IFG	−0.0649	0.3206
	Descending	Left STG	Left A1	0.2443	0.3002
		Right STG	Right A1	0.1396	0.2769
		Left IFG	Left STG	0.1713	0.4291
		Right IFG	Right STG	−0.0327	0.3105
Half (second vs. first)	Intrinsic	Left A1	Left A1	0.0477	0.1322
		Right A1	Right A1	0.0673	0.1268
		Left STG	Left STG	0.0192	0.1279
		Right STG	Right STG	0.0356	0.1139
		Left IFG	Left IFG	0.0089	0.1403
		Right IFG	Right IFG	0.0032	0.1388
	Ascending	Left A1	Left STG	−0.1463	0.2264
		Right A1	Right STG	0.0623	0.2338
		Left STG	Left IFG	0.0683	0.3223
		Right STG	Right IFG	−0.0077	0.258
Block (late vs. early)	Ascending	Left A1	Left STG	0.0207	0.221
		Right A1	Right STG	−0.0614	0.2263
		Left STG	Left IFG	−0.0622	0.3089
		Right STG	Right IFG	−0.0519	0.2918
	Descending	Left STG	Left A1	−0.0084	0.2099
		Right STG	Right A1	−0.0356	0.2262
		Left IFG	Left STG	0.0657	0.3351
		Right IFG	Right STG	0.0914	0.3048
Sequence (late vs. early)	Intrinsic	Left A1	Left A1	−0.0769	0.1354
		Right A1	Right A1	−0.0702	0.126
		Left STG	Left STG	−0.0118	0.1207
		Right STG	Right STG	−0.0842	0.1237
		Left IFG	Left IFG	−0.0008	0.1362
		Right IFG	Right IFG	0.0084	0.1208
	Ascending	Left A1	Left STG	−0.0642	0.1669
		Right A1	Right STG	0.2232	0.1488
		Left STG	Left IFG	0.095	0.1851
		Right STG	Right IFG	0.021	0.1784
	Descending	Left STG	Left A1	0.0401	0.1746
		Right STG	Right A1	0.1134	0.1739
		Left IFG	Left STG	−0.2224	0.1938
		Right IFG	Right STG	−0.1038	0.1921

*Note:* Positive (vs. negative) posterior means indicate increased (vs. decreased) connectivity strength due to contextual factors or multi‐timescale dynamics. An exponent of the posterior mean corresponds to the proportion of the connectivity weight in one condition relative to another—e.g., exp(−0.0689) = 93.34% for original deviants (relative to 100% for reverse deviants), amounting to a 6.66% connectivity decrease.

#### 3.2.1 Effects of Deviant Type

**FIGURE 4:**
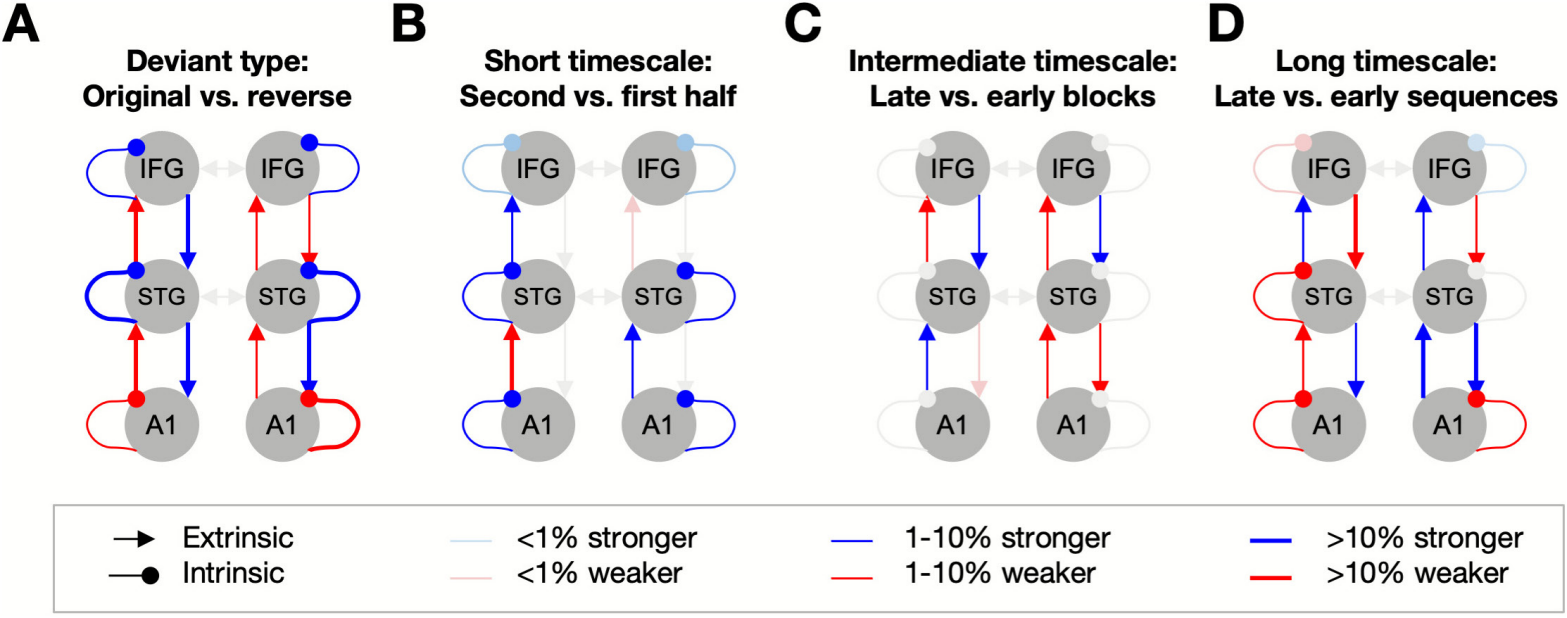
DCM results (independent of age). (A) Effects of deviant type. Connectivity modulation for original deviants, relative to reverse deviants. Blue: net excitation, red: net inhibition. Thick arrows: > 10% connection weight change between conditions, thin arrows: 1%–10% connection weight change, pale arrows: < 1% connection weight change. (B) Effects of the short timescale. Connectivity modulation for the second half of each block, relative to the first half. Legend as above. (C) Effects of the intermediate timescale. Connectivity modulation for the second block of each type, relative to the first block. Legend as above. (D) Effects of the long timescale. Connectivity modulation for the later sequences, relative to the earlier sequences. Legend as above.

Compared to the reversed deviant, the original deviant elicited a significant decrease in ascending connectivity across the network, with reductions in A1 → STG and STG → IFG. Descending connectivity increased bilaterally throughout the network, with the exception of right IFG → STG, which decreased its strength. These findings suggest a stronger reliance on predictions and a reduction in prediction error signaling for the original deviant. Additionally, intrinsic connectivity increased in STG and IFG but decreased in A1, reflecting modulation of precision weighting at different processing levels. These connectivity changes are visualized in Figure 4A, depicting the relative differences in effective connectivity for original versus reversed deviants, consistent with higher precision at higher levels.

#### 3.2.2 Effects of Timescales

At the shortest time scale, intrinsic connectivity increased throughout the network for the second versus first half of each block, reflecting an overall increase in ERP amplitude. This was accompanied by a more heterogeneous pattern of modulations of ascending connections (increased weight of right A1 → STG and left STG → IFG, and decreased weight of left A1 → STG and right STG → IFG). Therefore, the net ascending drive in the STG showed a right‐hemispheric shift. These connectivity changes are visualized in Figure 4B.

At the intermediate timescale, neural responses to late‐block deviants, compared to early‐block deviants, were associated with increased descending connectivity at higher levels of the cortical hierarchy. The IFG → STG connection strengthened (and the STG → IFG connection weakened) bilaterally, suggesting an increased influence of prior expectations as blocks progressed. At the lower levels, this was compensated by descending connectivity from STG to A1 decreasing bilaterally, and an asymmetric modulation of ascending connectivity (increasing in the left and decreasing in the right hemisphere). Therefore, the net ascending drive in the STG showed a left‐hemispheric shift. The relative connectivity changes for early versus late blocks are shown in Figure 4C.

At the longest timescale, the modulation of connectivity followed a distinct pattern. Ascending connectivity increased throughout the network, except the left‐hemispheric connection between A1 and STG. Top‐down connectivity from STG to A1 increased, while connectivity from IFG to STG decreased bilaterally, possibly reflecting a shift from higher‐order predictive control to local adaptation at earlier cortical stages. Intrinsic connectivity decreased in all regions except for a slight increase in the right IFG, which may indicate a refinement of precision weighting mechanisms as the task progressed. The relative connectivity changes between early and late sequences are depicted in Figure 4D.

#### 3.2.3 Effects of Age

In the second part of the DCM analysis, we looked at the modulatory effect of age on three groups of connections (ascending, descending, intrinsic), which could be modulated by each of the two significant effects we found at the sensor level (related to differences between deviant type and the longest time scale). In this case, the full model space consisted of 2^2*3^ = 64 models per participant, which were analyzed as above. The winning model (model probability *p* = 0.57 out of all 64 models) allowed for a subset of connections to be modulated by age (deviant type connectivity: only ascending and descending connections; long timescale connectivity: only descending connections). However, given that the winning model did not yield *p* > 0.95 probability, models were subjected to BMA, whereby parameter estimates were weighted by model evidence. Table 2 provides a full summary of all parameters at the group level after BMA.

**TABLE 2:** Age effects on DCM connectivity estimates. Positive (vs. negative) posterior means indicate increased (vs. decreased) connectivity strength with age.

Effect	Connection type	From	To	Posterior mean	Variance (×10^−3^)
Age × deviant type (original vs. reverse)	Ascending	A1 left	STG left	−0.0919	0.2947
		A1 right	STG right	−0.0051	0.2915
		STG left	IFG left	0.0608	0.4221
		STG right	IFG right	0.0893	0.3292
	Descending	STG left	A1 left	0.0574	0.2915
		STG right	A1 right	0.0048	0.3049
		IFG left	STG left	0.0468	0.4258
		IFG right	STG right	0.0981	0.3311
Age × sequence (late vs. early)	Descending	STG left	A1 left	−0.0715	0.1829
		STG right	A1 right	−0.0294	0.1736
		IFG left	STG left	−0.0397	0.2139
		IFG right	STG right	0.0758	0.206

**FIGURE 5:**
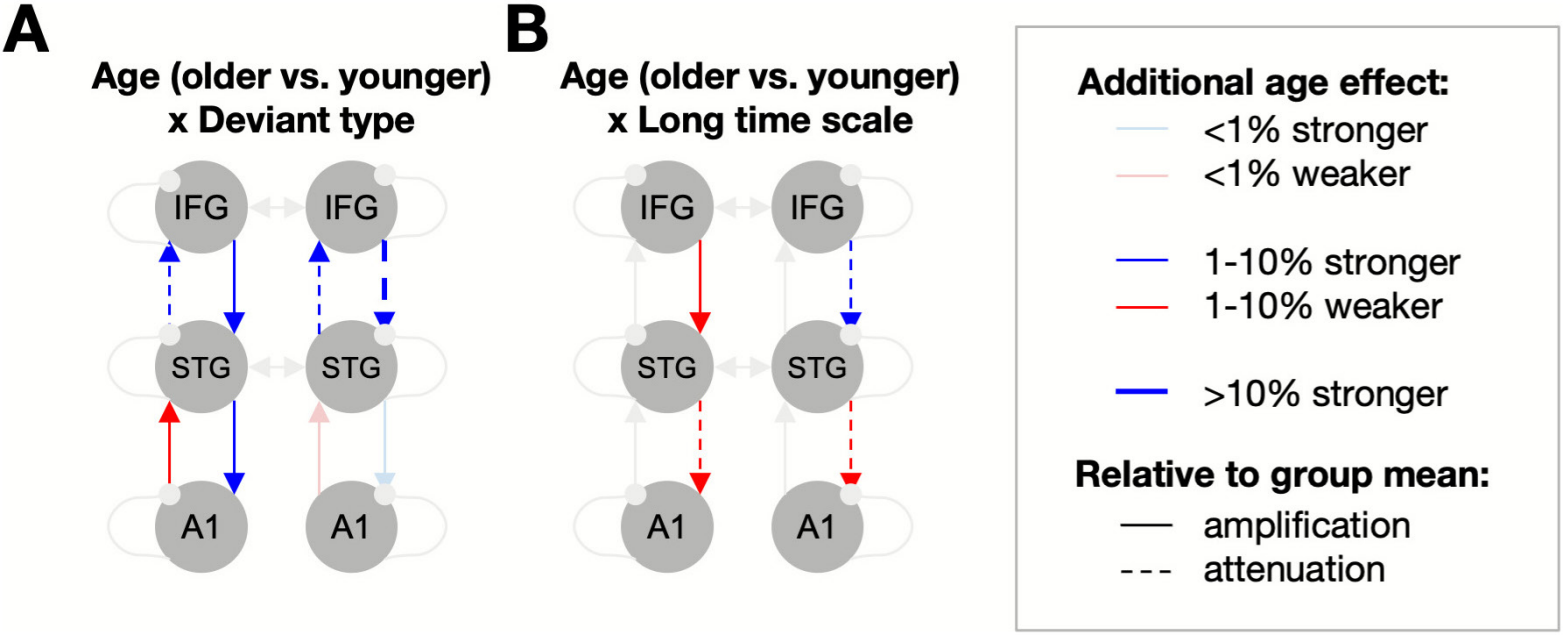
DCM results (effects of age). (A) Age effects on connectivity modulation by deviant type. The plot shows additional age‐related modulation for original deviants, relative to reverse deviants. Blue: net age‐related excitation, red: net age‐related inhibition. Thick arrows: > 10% connection weight change between conditions, thin arrows: 1%–10% connection weight change, pale arrows: < 1% connection weight change. Solid arrows: augmented connectivity (age effects consistent with mean effects, see Figure 4A); dashed arrows: attenuated connectivity (age effects opposing mean effects). (B) Age effects on connectivity modulation by the long timescale. The plot shows additional age‐related modulation for late sequences, relative to early sequences. Legend as above.

With increasing age, all descending connections which mediated different deviant types (Figure 5A) became stronger for original versus reverse deviants, while ascending connections became stronger for original deviants at higher levels of the hierarchy (STG → IFG) and weaker at lower levels of the hierarchy (A1 → STG). A comparison between the additional effects of age (Figure 5A) and the group mean (Figure 4A) revealed that connectivity at higher levels of the hierarchy (between STG and IFG; except the left descending connection) was relatively attenuated in older participants—i.e., the difference between original and reverse deviants was positive for the group mean but the additional age effect was negative. In contrast, connectivity at the lower levels of the hierarchy (between A1 and STG) became more pronounced in older participants (e.g., if the modulation by deviant type was positive for the group mean, it became even more positive with age, and vice versa).

Finally, with increasing age, all descending connections mediating the longest timescale (sequence number) became weaker for later versus earlier sequences (Figure 5B), with the exception of the right‐hemispheric IFG‐STG connection. In comparison to the group mean, this amounted to an overall age‐related reduction in connectivity modulation by the longest timescale (except the left descending connection, which became more inhibitory with age)

## 4 Discussion

This study investigated neural responses in a multi‐timescale oddball paradigm using two alternating auditory deviants. In the first step, ERP responses were analyzed at the sensor level, revealing significant effects of deviant type and each of the three timescales. Subsequently, DCM was applied to provide a mechanistic explanation of these differences in terms of effective connectivity. The results highlight how PP mechanisms shape auditory perception over time, how they change with age, and how initial stimulus expectations persist even when stimulus probabilities change.

### 4.1 Effects of Deviant Type

The current study showed significant differences in ERP responses between original and reversed deviants. Consistent with previous findings (Frost et al. 2016; Todd et al. 2021; Todd et al. 2011), deviant‐evoked ERP amplitudes were stronger for the original deviant, supporting the existence of a primacy effect—where the initial role assignment of a tone continues to influence later responses. This suggests that even after tone probabilities are inverted, remnants of the initial internal model persist, modulating subsequent perception. The current study replicates previous findings (Frost et al. 2016; Todd et al. 2021; Todd et al. 2011) even though the tone type was reversed—i.e., here the short tone was the original and the long tone was the reverse deviant, while the previous studies had the opposite order.

More generally, this finding is compatible with results showing that when a new regularity emerges in the sound sequence, the model of the previous one is still effective for a while (i.e., deviant sounds elicit MMN with respect to it) even without encountering further sounds meeting the old regularity (Winkler, Karmos, and Näätänen 1996). Studies showing that a single regular (reminder) sound can reactivate a model that has become inactive (i.e., deviant sounds do not anymore elicit MMN) also support the persistence of internal auditory models (Winkler, Karmos, and Näätänen 1996; Winkler and Cowan 2005). The relatively long time needed for eliminating established models is in sharp contrast to the speed of learning new models (Bendixen et al. 2007), probably because retaining models allows for quick adaptation to recurring auditory regularities (Winkler and Schröger 2015).

DCM analysis provided insight into the underlying neural mechanisms of this model persistence. Compared to the reversed deviant, original deviant responses were associated with increased top‐down connectivity and decreased bottom‐up connectivity. This suggests that perception of the original deviant relied more heavily on predictions, reducing the impact of incoming sensory input. This aligns with previous DCM studies on MMN, which found that increased reliance on prior expectations corresponds to stronger top‐down influences and reduced bottom‐up signaling (Fitzgerald et al. 2021). Additionally, intrinsic connectivity increased in STG and IFG but decreased in A1, consistent with modulations of precision weighting at different cortical levels (Moran, Campo, et al. 2013).

### 4.2 Effects of Timescales

In addition to deviant type, temporal factors influenced neural processing at multiple timescales. At the shortest timescale, ERP amplitudes increased within blocks. DCM linked these effects to an overall increase in connectivity—both ascending, putatively linked to prediction error processing (Bastos et al. 2012), and intrinsic, linked to postsynaptic gain modulation. The connectivity effect sizes were strongest at the lower level of the hierarchy, including A1 and STG. In the context of PP theories, increased gain and prediction error signaling is usually explained in terms of higher precision weighting of prediction errors (Bastos et al. 2012; Auksztulewicz and Friston 2015). Intrinsic connectivity has been previously shown to be subject to fast modulatory effects, potentially linked to short‐term plasticity (Garrido, Kilner, Kiebel, Stephan, et al. 2009). This mechanism may underlie what is traditionally conceptualized in psychology as the gradual sharpening of auditory memory traces (i.e., models of auditory regularities), as was shown by MMN studies testing the learning of complex sound patterns (Näätänen et al. 1993).

In contrast, at the intermediate timescale, ERP amplitudes decreased between blocks of the same type (separated by alternate blocks). This effect was linked using DCM to a distinct pattern of connectivity modulation, relying only on extrinsic connections. Specifically, most ascending connections decreased, and most descending connections increased in late versus early blocks. This may suggest that as exposure to a stimulus continues, perception becomes more reliant on prior knowledge (mediated via descending connections). This interpretation is consistent with PP models, which propose that prediction errors decrease over time as internal models become more accurate (A. Clark 2013). Reduced MMNs have been previously observed by the end of longer stimulus blocks (Winkler, Cowan, et al. 1996), an effect that might stem from the same source.

At the longest timescale, ERP amplitudes also gradually declined across sequences (separated by short silent periods). However, these changes were linked to a different pattern of connectivity modulations than in the case of the intermediate timescale. Changes across sequences involved both intrinsic and extrinsic connections. The intrinsic connections showed the opposite pattern of modulation than at the shortest timescale, decreasing (rather than increasing) over time. Furthermore, the extrinsic connections showed the opposite pattern of modulation than at the intermediate timescale, overall increasing (rather than decreasing) ascending drive. Additionally, descending connectivity increased at lower levels of the hierarchy (from STG to A1) and decreased at the higher levels of the hierarchy (from IFG to STG). Prior research suggests that precision weighting increases when an environment is perceived as stable and predictable (Fitzgerald et al. 2021). The observed reduction in intrinsic connectivity may therefore reflect an overall drop of precision following interruptions between continuous sequences, which may induce surprise and trigger relearning. The gradual shift of increased descending connectivity from higher levels of the hierarchy at intermediate timescales to lower levels of the hierarchy at longer timescales is consistent with the theory of reverse hierarchies in sensory learning, which suggests that, at longer timescales, learning effects are expressed in lower‐level regions, akin to perceptual (rather than statistical) learning (Ahissar et al. 2009; Fiser and Lengyel 2019). This is consistent with the notion that, following prolonged learning, predictions are delegated to hierarchically lower regions, while frontal cortices are effectively disengaged.

Finally, the deviant type interacted with the short and intermediate timescale. Within blocks (short timescale), original deviants evoked stronger ERPs than reverse deviants already in the first half of each block, but in the second half this difference gradually diminished. Across blocks (intermediate timescale), ERPs evoked by original deviants gradually decreased, while ERPs evoked by reverse deviants remained stable. Taken together, this is consistent with a rapid initial boost in precision‐weighting for original deviants—i.e., the primacy effect (Todd et al. 2011)—which may be partly reduced as the auditory system accumulates more evidence at different timescales.

Although interactions between deviant type and temporal factors were significant in ERP analysis, these interactions were not explicitly modelled in DCM. This is due to the nonlinear nature of DCM equations, which allow main effects to account for variance that would otherwise be attributed to interactions (Auksztulewicz and Friston 2015). However, it remains possible that distinct predictive mechanisms operate at different learning stages, as seen in other studies (McDermott et al. 2024). Future research could explore whether early and late learning stages are associated with qualitatively different connectivity patterns.

### 4.3 Effects of Age

Older participants showed reduced deviant‐evoked responses and reduced adaptation at the longest timescale, consistent with recent reports of general age‐related deficits in basic auditory processing (Criscuolo et al. 2025) as well as more specific impairments in temporal integration at longer time scales (Todd et al. 2022). Additionally, our ERP analysis indicated that the difference between original and reverse deviant responses was modulated by age, such that older participants showed a distinct ERP waveform for reverse deviants—peaking and rebounding faster than the waveform for original deviants in older participants, as well as both deviant waveforms in younger participants. Similar ERP results have been found for a subset of the dataset analyzed in a previous study (Todd et al. 2022).

Connectivity analyses revealed that, unlike at the level of the group mean (independent of age) where the longest timescale affected all types of connections (intrinsic, ascending, and descending), the additional effect of age was confined to descending connections. More specifically, the interaction between age and deviant type could be linked to an overall age‐related decrease in connectivity modulation by deviant type. This relative attenuation was most pronounced at the lower levels of the hierarchy. Such age‐related impairments, amounting to weaker modulation of low‐level descending connectivity, may therefore underlie deficits in learning slowly evolved sequence statistics, consistent with previous reports of age deficits in perceptual learning (Alain and Snyder 2008), while sequence statistics that can be extracted quickly may remain relatively intact (Boncz et al. 2024).

Deviant type effects were similarly modulated by age. Again, age‐related effects were more confined than age‐independent effects: whereas in general original deviants modulated all types of connections, the effect of age was confined to extrinsic connections. With age, the modulatory effects of deviant type became relatively augmented at the lower levels of the hierarchy but attenuated at the higher levels of the hierarchy. These results are partly consistent with previous DCM studies on age‐related auditory processing (Moran et al. 2014; Cooray et al. 2014), albeit those studies used classical oddball designs rather than multi‐timescale sequences. Similar to the former study (Moran et al. 2014), we found that older participants showed net inhibition of the ascending connectivity from A1 to STG. In contrast, unlike the latter study, which reported impaired intrinsic IFG connectivity in the right hemisphere (Cooray et al. 2014), in our results, the effects of age were confined to extrinsic connections. However, similar to that study, we found consistent age‐related attenuation of right‐hemispheric IFG connectivity, which affected both the processing of deviant types and of the longest timescale. Therefore, our study generalizes those previous findings beyond classical MMN to more contextually rich and dynamic sequences.

In the broader context of normative electrophysiological aging, our findings align with well‐established patterns of spectral changes across the lifespan. Previous studies have shown that alpha power and peak frequency decline with age, while delta and theta power increase (Babiloni et al. 2011; Rossini et al. 2007). Beta power tends to increase in older adults (Inamoto et al. 2023; Karekal et al. 2023; Prichep et al. 1994; Rossiter et al. 2014), although some evidence suggests that this trend may be nonlinear, peaking in late middle age before declining again in later years (Dustman et al. 1999). While our primary focus was not on spectral dynamics—given that our analyses centered on ERP components, which, unlike induced oscillatory activity, are well‐suited to connectivity modelling using biophysically plausible neural mass models—these shifts in baseline oscillatory activity may still influence the observed effects. Slower intrinsic rhythms and reduced signal‐to‐noise ratios could affect both the temporal precision of auditory predictions and the efficacy of top‐down modulation, potentially accounting for the attenuated descending connectivity and altered deviance responses observed in older adults. These functional changes may reflect broader age‐related neurobiological processes, such as altered communication between regions, which have been proposed to underlie diminished plasticity in aging (Voytek and Knight 2015).

## 5 Conclusions

The current study aimed to examine how temporal factors influence neural connectivity in response to alternating auditory oddball segments through the application of DCM. Building on previous research, the investigation focused on the primacy effect, which posits that initial role assignments of auditory tones can shape later neural responses across various timescales.

Overall, our findings demonstrate how predictive mechanisms shape auditory processing across contexts and multiple timescales. We also identify specific age‐related modulations of both the measured neural responses and their underlying putative connectivity mechanisms. The results emphasize the brain's capacity for flexible adaptation, where early stimulus expectations persist despite changing contexts, and long‐term exposure shifts reliance on top‐down versus bottom‐up processing. By identifying distinct patterns of connectivity modulation, this study provides further evidence that auditory learning is dynamically regulated by interactions between prediction errors, priors, and precision weighting.

## Author Contributions

**Martin Tom Banaschewski:** formal analysis, visualization, writing – original draft, writing – review and editing. **Christoph Mathys:** conceptualization, funding acquisition, writing – original draft, writing – review and editing. **István Winkler:** conceptualization, funding acquisition, writing – original draft, writing – review and editing. **Juanita Todd:** conceptualization, data curation, formal analysis, funding acquisition, investigation, project administration, supervision, writing – original draft, writing – review and editing. **Ryszard Auksztulewicz:** conceptualization, formal analysis, methodology, supervision, visualization, writing – original draft, writing – review and editing.

## Funding

This work was supported by Australian Research Council (DP200102346), Hungarian National Research, Development and Innovation Office (K132642), Carlsberg Foundation (CF21‐0439), Deutsche Forschungsgemeinschaft (AU 423/2‐1), Dutch Research Council (406.22.24GO.030), Aarhus Universitets Forskningsfond (AUFF‐E‐2019‐7‐10), and Danmarks Frie Forskningsfond (3166‐00158B).

## Ethics Statement

The study was approved by the Human Research Ethics Committee of the University of Newcastle.

## Conflicts of Interest

The authors declare no conflicts of interest.

## Data Availability

Data and scripts will be uploaded to a public repository upon manuscript acceptance.
